# Extracellular CIRP decreases Siglec-G expression on B-1a cells skewing them towards a pro-inflammatory phenotype in sepsis

**DOI:** 10.1186/s10020-021-00318-y

**Published:** 2021-05-31

**Authors:** William Royster, Hui Jin, Ping Wang, Monowar Aziz

**Affiliations:** 1 Center for Immunology and Inflammation, The Feinstein Institutes for Medical Research, 350 Community Dr, Manhasset, NY 11030 USA; 2 Elmezzi Graduate School of Molecular Medicine, Manhasset, NY 11030 USA; 3 Department of Surgery, Donald and Barbara Zucker School of Medicine At Hofstra/Northwell, 350 Community Dr, Manhasset, NY 11030 USA; 4 Department of Molecular Medicine, Donald and Barbara Zucker School of Medicine At Hofstra/Northwell, Manhasset, NY 11030 USA

**Keywords:** eCIRP, B-1a cells, Siglec-G, TLR4, Sepsis

## Abstract

**Background:**

Sepsis is a life-threatening disease syndrome caused by a dysregulated host response to infection and injury. Extracellular cold-inducible RNA-binding protein (eCIRP) acts as a damage-associated molecular pattern. Peritoneal cavity (PerC) B-1a cells attenuate inflammation and tissue injury by spontaneous releasing natural IgM and IL-10. Sialic acid-binding immunoglobulin-type lectin-G (Siglec-G) is a CD33-related receptor highly expressed in B-1a cells to serve critical immunoregulatory functions. In sepsis, B-1a cell numbers in PerC are decreased. We hypothesized that eCIRP causes the reduction of PerC B-1a cells and alters their function during sepsis.

**Methods:**

Sepsis was induced in WT and CIRP^−/−^ mice by cecal ligation and puncture (CLP). PerC washout cells were collected and B-1a cells and Siglec-G were assessed by flow cytometry. Mice were *i.p.* injected with recombinant murine (rm) CIRP and after 20 h, Siglec-G expression in PerC B-1a cells were assessed. PerC B-1a cells were treated with rmCIRP for 4 h and Siglec-G expression was assessed. PerC B-1a cells were pre-treated with anti-Siglec-G Ab and then after stimulated with rmCIRP for 24 h, IL-6 levels in the culture supernatants were assessed.

**Results:**

eCIRP levels in the PerC were elevated in septic mice. In WT mice, the frequencies and numbers of total and Siglec-G^+^ B-1a cells in the PerC were significantly decreased in the CLP group compared to sham group, whereas in CIRP^−/−^ mice, their frequencies and numbers in sepsis were significantly rescued compared to WT septic mice. Mice injected with rmCIRP showed decreased frequencies and numbers of total and Siglec-G^+^ PerC B-1a cells compared to PBS-injected mice. In vitro treatment of PerC B-1a cells with rmCIRP demonstrated significant reduction in Siglec-G mRNA and protein compared to PBS group. PerC B-1a cells treated with anti-Siglec-G Ab had significantly higher production of IL-6 in response to rmCIRP compared to IgG control. Anti-Siglec-G Ab treated B-1a cells co-cultured with macrophages produced significantly higher levels of IL-6, and TNF-α, and lower levels of IL-10 compared to IgG-treated B-1a cells and macrophage co-cultures stimulated with rmCIRP.

**Conclusion:**

eCIRP reduces PerC B-1a cell pool and skews them to a pro-inflammatory phenotype by downregulating Siglec-G expression. Targeting eCIRP will retain Siglec-G expressing B-1a cells in the PerC and preserve their anti-inflammatory function in sepsis.

**Supplementary Information:**

The online version contains supplementary material available at 10.1186/s10020-021-00318-y.

## Introduction

Sepsis is defined as a dysregulated host response to an infection, resulting in severe systemic inflammation, multisystem organ dysfunction, and ultimately death (Singer 2016). There are approximately 19.4 million cases of sepsis annually and nearly 5.3 million deaths (Rudd 2020). Sepsis is initiated by simultaneous recognition of pathogen-associated molecular patterns (PAMPs) and endogenous damage-associated molecular patterns (DAMPs) by the pattern-recognizing receptors (PRRs) on immune cells. Binding of PAMPs or DAMPs to PRRs induces activation of downstream pathways, leading to increased expression of the mediators of inflammation (Aziz et al. 2013).

Cold-inducible RNA-binding-protein (CIRP) is an 18-kDa nuclear RNA chaperone protein (Nishiyama 1997). During inflammation and sepsis, intracellular CIRP (iCIRP) has been shown to be released extracellularly both passively after cellular necrosis and actively by lysosomal-exocytosis (Qiang 2013). Elevated plasma levels of extracellular CIRP (eCIRP) have been correlated with poor outcomes in patients suffering from sepsis (Qiang 2013; Zhou 2015). eCIRP promotes activation of multiple different cell types, including macrophages, T lymphocytes, and neutrophils, subsequently potentiating cytokine and chemokine production by recognizing Toll-like receptor 4 (TLR4) and triggering receptor expressed on myeloid cells-1 (TREM-1) (Aziz et al. 2019; Denning 2020). The impact of eCIRP on regulatory B lymphocytes such as B-1a cells in sepsis has never been studied.

B-1 cells are a subtype of B lymphocytes. Based on the presence or absence of the CD5 surface marker, B-1 cells can be further divided into B-1a (CD5^+^), and B-1b (CD5^−^) cells. The expression of other identifying surface markers B220^lo^, CD23^lo/−^, CD19^hi^, CD43^+^ and CD11b^+^ remain similar in both subtypes of B-1 cells (Aziz et al. 2015; Rothstein et al. 2013). B-1a cells play a vital role in the initial defense against invading pathogens by spontaneously releasing germline-like natural IgM (Grönwall et al. 2012; Boes et al. 1998). B-1a cells produce the anti-inflammatory cytokine IL-10 spontaneously as well as in response to inflammatory stimulation (Aziz et al. 2017). These cells also release granulocyte-monocyte colony-stimulating factor (GM-CSF), which in turn promotes a more robust release of natural IgM by the B-1a cells, serving to protect the host from invading pathogens (Rauch 2012; Weber 2014). Conversely, B-1a cells also produce IL-3, IL-17, TNF-α, and IL-6 which exhibit pro-inflammatory roles in sepsis (Aziz et al. 2015, 2017; Weber 2015). Previous work has demonstrated that B-1a cells contribute to improved outcomes in bacterial sepsis, pneumonia, and viral infection. We have recently revealed that the B-1a cell numbers are significantly decreased in sepsis, and treatment of septic mice with B-1a cells ameliorates systemic inflammation and acute lung injury (ALI) (Aziz et al. 2017; Aziz 2018). Intraperitoneal injection of mice with lipopolysaccharide (LPS) results in a significant decrease of B-1a cell pool in the peritoneal cavity (PerC) and promotes their prompt migration to the spleen and lymph nodes (Aziz et al. 2017; Moon et al. 2012). However, it remains unknown whether the new DAMP, eCIRP is capable of exhibiting a similar effect on peritoneal B-1a cells.

Sialic acid-binding immunoglobulin-type lectin-G (Siglec-G), a CD33-related receptor highly expressed in B-1a cells, serves important immunoregulatory functions (Royster et al. 2021). Dendritic cells, macrophages, and T lymphocytes have also been shown to express Siglec-G, but to a reduced level. The sialic acid binding domain is located on the N-terminal Ig-like domain of Siglec-G. The other parts of Siglec-G include a transmembrane region and an intracellular tail which contains an immunoreceptor tyrosine-based inhibitory motif (ITIM), an ITIM-like domain, and a Grb-2-binding site (Royster et al. 2021). Siglec-G binds to sialic acid moieties on B cell receptor (BCR), thereby blocking BCR-mediated downstream signaling (Jellusova and Nitschke 2011). In myeloid cells, Siglec-G acts as a negative regulator of DAMPs. CD24, a heavily sialylated glycoprotein, directly binds multiple different DAMPs, which then in turn binds to Siglec-G forming a trimeric complex to repress the inflammatory signal cascade in response to DAMPs (Chen et al. 2009; Chen 2011). Thus, conserving Siglec-G’s function in immune cells protects mice from inflammation.

In the current study, we aimed to identify the direct impact of eCIRP on murine peritoneal B-1a cells and assess the expression of Siglec-G in peritoneal B-1a cells in sepsis. Our data reveal that eCIRP significantly reduces the PerC B-1a cell pool and skews B-1a cells to a pro-inflammatory phenotype by decreasing Siglec-G expression. Hence, targeting eCIRP could help B-1a cells remain in the peritoneal cavity and preserve their anti-inflammatory function in sepsis.

## Materials and methods

### Experimental animals

Wild-type (WT) male, C57BL/6 mice (8–12 weeks old, 23–27 g BW) were purchased from Charles River Laboratories (Kingston, NY). C57BL/6 CIRP^−/−^ mice were obtained from Dr. J. Fujita (Kyoto University, Kyoto, Japan) as a kind gift. Siglec-G^−/−^ mice were generated with assistance from Dr. L. Li at Cold Spring Harbor Laboratory (Cold Spring Harbor, NY) utilizing CRISPR/Cas-9 transfection of guide RNA (gRNA) designed under research contract by Transviragen (Chapel Hill, NC) to target deletion of exons 2–3 of the murine Siglec-G gene on C57BL/6 background mice. Briefly, gRNA sequences were cloned into T7 expression vectors, sequence-validated gRNAs were generated by in vitro transcription. gRNA/Cas-9 ribonucleoprotein complexes were transfected into a mouse embryo fibroblast (MEF) cell line and lysates were harvested 72 h later for DNA isolation. PCR amplification and sequencing of gRNA cut site regions was performed followed by Synthego Inference of CRISPR Edits (ICE) analysis to quantitatively assess gRNA activity. Siglec-G Intron-1 gRNA (TTGTCTTGCACCGGTACCC) and Intron 3 gRNA (GTAAGAGTTGTAAGACATAGC) were noted to have the highest ICE activity scores, at 17 and 13 respectively, with the best predicted off target activity. Mice were housed in temperature-controlled environments and fed a diet of standard laboratory mouse chow. Mice were allowed *ad lib* access to food and water throughout the experiment. The mice were kept on a 12 h light/dark cycle. All animal experiments were performed in accordance with the National Institutes of Health guidelines for the care and use of laboratory animals. This study was approved by the Institutional Animal Care and Use Committee of the Feinstein Institutes for Medical Research.

### Cecal ligation and puncture (CLP) model

Mice were made septic by CLP, following the protocol of our previous study (Aziz et al. 2017). Mice were anesthetized with 2% inhalational isoflurane and placed in a supine position. Hair was removed from the abdomen with clippers and the surgical field was prepped with isopropyl alcohol wipes and 10% povidone-iodine wash. A 2 cm midline laparotomy was performed. The cecum was eviscerated and ligated with 4–0 silk suture 1 cm proximal to the end of the cecum. The cecum was punctured twice with a 22-gauge needle 0.5 cm distal to the suture ligation and a small amount of feculent material was extruded out of the punctures. The cecum was returned to the abdominal cavity and the wound was closed in layers. All mice were resuscitated with a subcutaneous bolus of 0.5 mL of normal saline and monitored for recovery from anesthesia. Mice were returned to their cages and euthanized by CO_2_ inhalation 20 h after CLP. Peritoneal lavage samples were obtained by instilling 7–8 mL of PBS supplemented with 2% heat inactivated fetal bovine serum (FBS, MP Biomedicals, Irvine, CA) into the PerC. The abdomen was gently agitated and the lavage was aspirated. This process was repeated a second time, collecting a total of 15 mL of PerC sample. Sham mice underwent laparotomy with the same anesthesia time as the experimental animals without cecal manipulation and underwent tissue collection at the same time samples were collected from experimental groups. The CLP model tends to be variable from one lab to the next. Besides mice strains, age, and gender, cecal ligation at what length from the tip of the distal part of the cecum, number of punctures, and size of the needle are the critical factors for the difference in findings of CLP model that varies from lab to lab. As depicted in our previous studies, using the same model of sepsis by CLP, after 20 h of CLP operation, we got considerably higher levels of all the systemic and lung pro-inflammatory cytokines and systemic tissue injury markers, increased lung injury scores, and cellular apoptosis (Aziz et al. 2017; Aziz 2018). Using this model, we got a 50% mortality rate of mice at 10-days after CLP operation with a single dose of antibiotic administration subcutaneously (Aziz et al. 2017).

### In vivo administration of rmCIRP

Mice were injected i.p. with rmCIRP (5 µg/kg BW) and then returned to their cages. At 20 h after i.p. injection, mice were euthanized with CO_2_ asphyxiation. PerC lavage samples were collected as described above.

### Isolation of B-1a cells

Mice were euthanized with CO_2_ asphyxiation. PerC lavage samples were collected as described above. PerC lavage was centrifuged at 300 × g for 10 min and re-suspended in PBS with 0.5% bovine serum albumin and 2 mM EDTA buffer. B-1a cells were isolated from PerC samples in accordance with manufacturer recommendations for MACS B-1a Cell Isolation Kit (Miltenyi Biotec, Bergisch Gladbach, Germany).

### Assessment of B-1a cells in PerC by flow cytometry

PerC B-1a cells were determined by flow cytometry as described previously (Aziz et al. 2017). PerC lavage samples were centrifuged at 300 × g for 10 min and re-suspended in 500 μL of FACS buffer. A total of 1 × 10^6^ PerC cells were suspended in 500 μL of FACS buffer and stained with PerCP-Cy5.5 anti-mouse CD5 antibody (clone: 53–7.3 BioLegend, San Diego, CA), PE anti-mouse CD23 antibody (clone: B3B4, BioLegend, San Diego, CA), FITC anti-mouse B220 antibody (clone: RA3-6B2, BD Biosciences, San Jose, CA), and APC anti-mouse Siglec-G antibody(clone: SH2.1, Invitrogen, Carlsbad, CA) for 30 min at 4 °C. Unstained cells were used to establish control voltage settings and single color compensation was established with UltraComp eBeads (Thermo-Fisher, Waltham, MA). Acquisition was performed on 100,000 events using a BD LSRFortessa flow cytometer (BD Biosciences, San Jose, CA) and data were analyzed with FlowJo software (Tree Star, Ashland, OR). To ensure rigorous data analysis, we precisely gated the cells in FSC and SSC to exclude debris or dead cells, considering that apoptotic cells/debris are located at the proximity to the X- and Y-axis. We removed doublets using FSC-A/FSC-H gating, which is a commonly used method for the exclusion of doublets.

### Isolation of PerC macrophages and co-culture with B-1a cells

WT mice were euthanized with CO_2_ inhalation. PerC lavage were collected as previously described and centrifuged at 300 × g for 10 min. The resulting pellet was suspended in RPMI 1640 (Invitrogen) supplemented with 10% FBS. Peritoneal macrophages were then allowed to adhere in a 10-cm culture plate for 2 h at 37 °C in 5% CO_2_. Non-adherent cells were removed by washing with pre-warmed culture medium. Adhered PerC macrophages were then mechanically detached from the plate using a rubber scraper and counted. Approximately, 1 × 10^5^ PerC macrophages were added to a 48 well flat-bottom cell culture plate. An equal amount of PerC B-1a cells were pretreated with 10 µg/mL of anti-Siglec-G Ab (clone: 805,903, R&D Systems, Minneapolis, MN) or 10 µg/mL of IgG control (Ultra-LEAF Purified Mouse IgG1 k isotype control clone: MG1-45, BioLegend) for 2 h at 4 °C. The Ab treated B-1a cells were centrifuged at 300 × g for 10 min to remove any excess Ab and the cells were added to the 48 well plate with the macrophages. The cocultured cells were then stimulated with 1 µg/mL rmCIRP or PBS control and the supernatant was collected after 24 h of incubation at 37 °C in 5% CO_2_.

### Enzyme-linked immunosorbent assay (ELISA)

Supernatant IL-6, IL-10, and TNF-α were analyzed according to manufacturer’s (Biosciences, San Jose, CA) instructions. Briefly, 10 µL of culture supernatants isolated from co-cultured cells were diluted in assay diluents to a final volume of 100 µL, loaded into ELISA wells precoated with the appropriate capture antibody, and incubated for 60 min at room temperature. Wells were washed and 100 µL of HRP-labeled conjugate was added to each well and incubated for 60 min at room temperature. The wells were washed and 100 µL of detection reagent was added to each well and allowed to develop color for 30 min. The reaction was stopped with the addition of 50 µL of 2 N H_2_SO_4_. Finally, OD was measured at 450 nm wavelength and the concentrations of samples were calculated from the standard curve. eCIRP concentration was assessed in PerC lavage samples by ELISA in accordance with manufacturer’s instructions (Mouse CIRP /CIRBP ELISA Kit—LS-F16777, LSBio, Seattle, WA).

### Statistical analysis

All statistical analyses were performed and the figures were prepared with GraphPad Prism version 9.0 software (GraphPad Software, La Jolla, CA). Data are expressed as mean ± standard error of the mean. Comparisons between two groups were performed with a two-tailed Student’s t-test (parametric). Comparisons between multiple groups were analyzed using a one-way analysis of variance (ANOVA), followed by Student–Newman–Keuls (SNK) comparison test. The statistical significance was set at p < 0.05.

## Results

### Sepsis increases eCIRP levels in the peritoneal cavity fluid

**Fig. 1:**
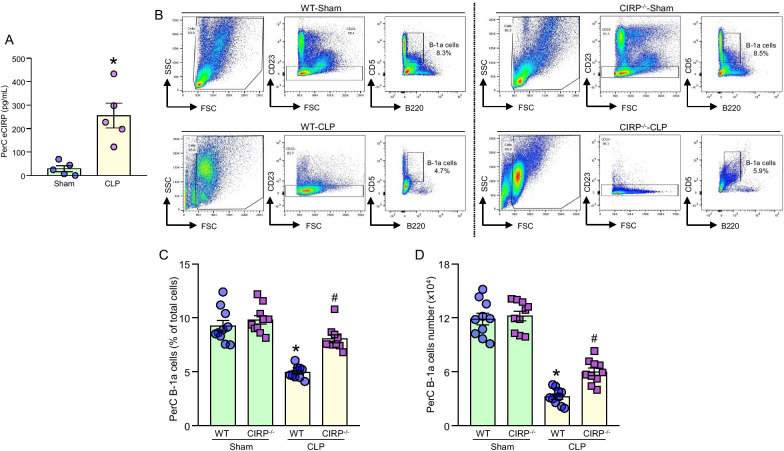
Intraabdominal sepsis results in a loss of PerC B-1a cells, while CIRP^−/−^ mice are protected from the loss of B-1a cells after sepsis. **A** After 20 h of CLP, peritoneal cavity lavage samples were collected. PerC lavage eCIRP concentration was assessed with ELISA. Data are expressed as means ± SE (n = 5–7 mice/group). The groups were compared by Student’s t-test (*p < 0.05 vs. sham mice). WT and CIRP^−/−^ mice were randomly assigned to sham or CLP groups. 20 h after surgery, PerC lavage samples were collected for analysis. **B** Representative dot blots of the gating strategy of B-1a cells of sham and CLP mice are shown. **C, D** Status of B-1a cell’s frequencies (%) and numbers after surgery are shown. Data are expressed as means ± SE (n = 10–14 mice/group). The groups were compared by one-way ANOVA and Student–Newman–Keuls (SNK) method (*p < 0.05 vs. WT sham and ^#^p < 0.05 vs. WT CLP mice)

Previously, we reported increased levels of eCIRP in serum and various organs in septic mice (Qiang 2013; Ode et al. 2018). Here, we assessed eCIRP levels in the PerC of septic mice. After 20 h of sepsis, peritoneal lavage fluid was collected to quantify PerC eCIRP levels. In sham mice, PerC eCIRP levels were quantified as 29 ± 13 pg/mL, while after sepsis was induced, the levels of PerC eCIRP were significantly increased to 256 ± 53 pg/mL, which was 8.8-fold higher than the sham levels (Fig. 1A).

### Deficiency of CIRP preserves peritoneal B-1a cell pool in sepsis

We induced sepsis in WT and CIRP^−/−^ mice and then assessed their B-1a cell contents in the peritoneal cavity. After 20 h of sepsis in WT mice, we noticed a significant decrease in the frequency and number of B-1a cells in the peritoneal cavity as compared to the WT sham mice (Fig. 1B–D). Interestingly, after 20 h of sepsis, the absolute decrease in the frequency of PerC B-1a cells in the CIRP^−/−^ mice was 19.5%, compared to an absolute decrease of 46.6% in WT septic mice (Fig. 1C). The absolute decrease in the number of PerC B-1a cells in CIRP^−/−^ septic mice was 50.3%, compared to an absolute decrease of 72.8% in the number of PerC B-1a cells in septic WT mice (Fig. 1D). These data suggest that eCIRP plays a pivotal role in decreasing PerC B-1a cell pool during sepsis.

### Deficiency of CIRP preserves Siglec-G’s expression in PerC B-1a cells in sepsis

**Fig. 2:**
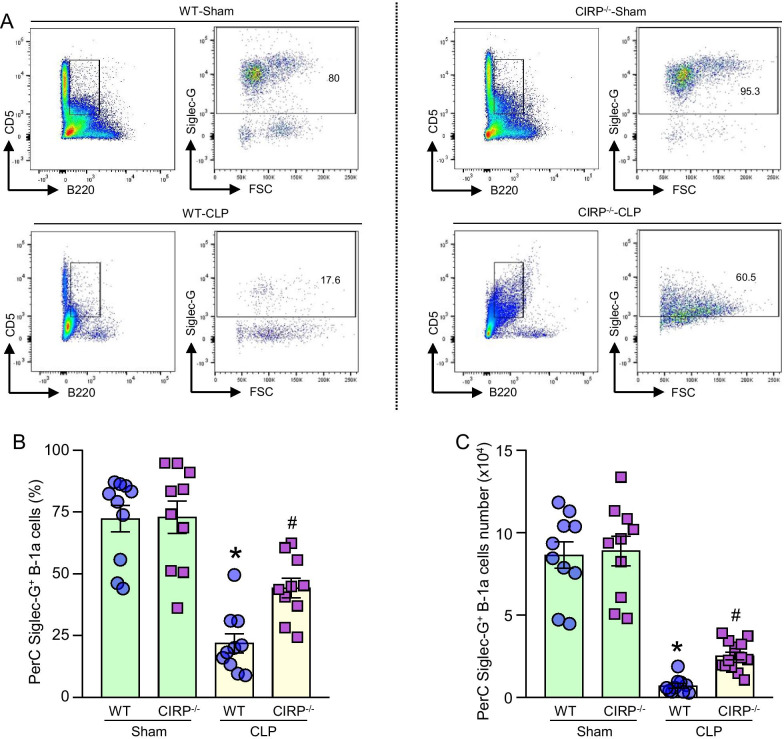
Expression of Siglec-G by B-1a cells is dramatically reduced after sepsis, but its expression is protected in CIRP^−/−^ mice after sepsis. WT and CIRP^−/−^ mice were randomly assigned to sham or CLP groups. 20 h after CLP, peritoneal lavage samples were collected for analysis. **A** Representative dot blots of the gating strategy of Siglec-G expression on B-1a cells of sham and CLP mice are shown. **B, C** Status of Siglec-G expression by B-1a cells in terms of frequency (%) and number after surgery are shown. Data are expressed as means ± SE (n = 10–14 mice/group). The groups were compared by one-way ANOVA and Student–Newman–Keuls (SNK) method (*p < 0.05 vs. WT sham and ^#^p < 0.05 vs. WT CLP mice)

Since Siglec-G plays an important role in controlling inflammation in immune cells (Chen et al. 2009), we next aimed to assess the expression of Siglec-G in PerC B-1a cells in sepsis. After 20 h of sepsis in WT mice, we noticed a significant decrease in the frequency and number of Siglec-G positive B-1a cells in the peritoneal cavity as compared to WT sham mice (Fig. 2A–C). Interestingly, after 20 h of sepsis, the absolute decrease in the frequency of Siglec-G positive PerC B-1a cells in the CIRP^−/−^ mice was 42.5%, compared to an absolute decrease of 69.8% in WT septic mice (Fig. 2B). The absolute decrease in the number of Siglec-G positive PerC B-1a cells in CIRP^−/−^ septic mice was 71.7%, compared to an absolute decrease of 91.8% in the number of PerC B-1a cells in septic WT mice (Fig. 2C). These data suggest that CIRP deficiency protects mice from the loss of Siglec-G positive PerC B-1a cells during sepsis.

### Treatment of healthy mice with rmCIRP decreases peritoneal B-1a cell pool

**Fig. 3:**
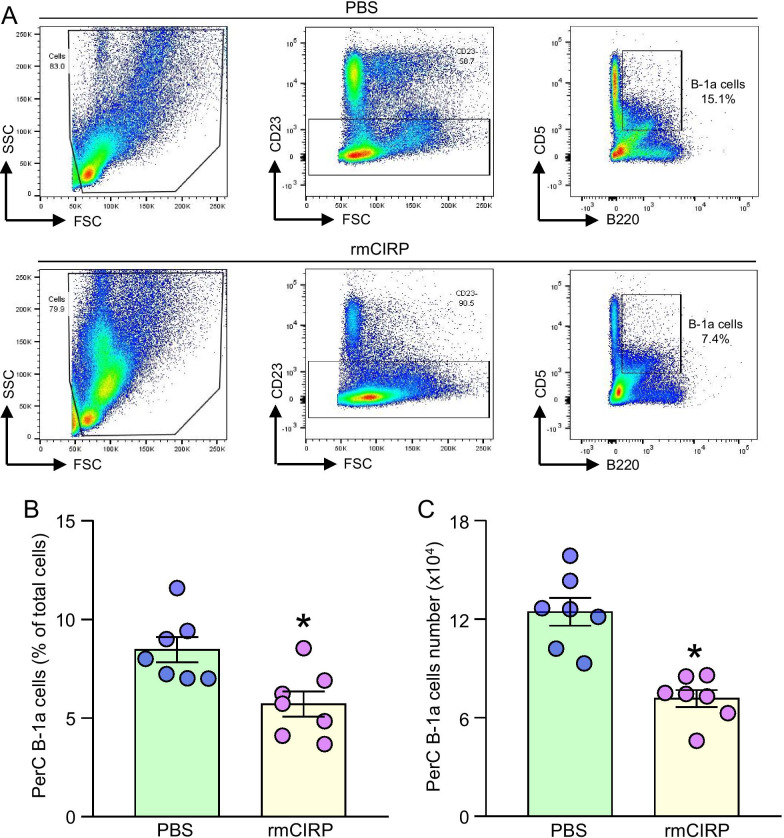
Intraperitoneal injection of rmCIRP results in a loss of B-1a cells from the PerC. **A** After 20 h of *i.p.* injection of rmCIRP in mice, PerC lavage samples were collected. Peritoneal cells were stained with CD23, B220, and CD5 Abs and subjected to flow cytometric detection of B-1a cells frequencies. Representative dot blots of the gating strategy of B-1a cells of vehicle and rmCIRP treated mice are shown. **B, C** Status of B-1a cell’s frequencies and numbers after *i.p.* injection of PBS vehicle or rmCIRP are shown. Data are expressed as means ± SE (n = 5–7 mice/group). The groups were compared by Student’s t-test (*p < 0.05 vs. vehicle-treated mice)

Mice were i.p. injected with rmCIRP. After 20 h of i.p. injection, peritoneal lavage fluid was collected and the frequency (%) and numbers of B-1a cells in the peritoneal cavity were assessed. In PBS-injected mice, the mean frequency of B-1a cells in the peritoneal cavity was 8.5%. However, treatment of mice with rmCIRP significantly decreased the PerC B-1a cell frequencies by 33.5% (Fig. 3A, B). In PBS-treated mice, the mean value of the numbers of B-1a cells in the peritoneal cavity was found to be 1.24 × 10^5^. Akin to the decrease in their frequencies, treatment of mice with rmCIRP significantly decreased the numbers of PerC B-1a cells by 44.2% (Fig. 3C). Thus, eCIRP decreases the B-1a cell pool in the peritoneal cavity.

### Treatment with rmCIRP reduces Siglec-G expression in the peritoneal B-1a cells in vivo and in vitro

**Fig. 4:**
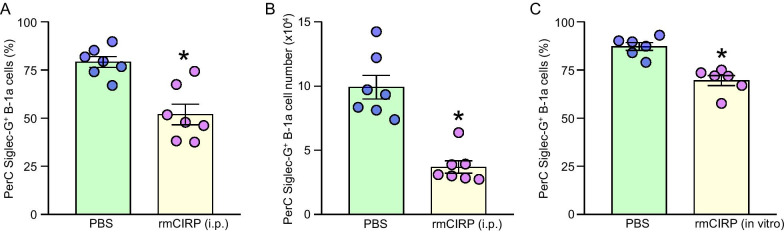
rmCIRP induces a downregulation of Siglec-G expression by B-1a cells in vivo and in vitro. 20 h after *i.p.* injection of rmCIRP, PerC lavage samples were collected. Peritoneal cells were stained with CD23, B220, CD5, and Siglec-G Abs and subjected to flow cytometric detection of B-1a cells and analysis of Siglec-G expression. Status of Siglec-G expression by B-1a cells in terms of **A** frequency and **B** number after *i.p.* injection of rmCIRP are shown. PerC B-1a cells were stimulated with rmCIRP in vitro for 24 h. Cells were stained with Siglec-G Ab and subjected to flow cytometric analysis. **C** Frequency of Siglec-G expression on B-1a cells after stimulation with rmCIRP are shown. Data are expressed as means ± SE (n = 6–7 mice/group). The groups were compared by Student’s t-test (*p < 0.05 vs. vehicle treatment)

To confirm the direct effect of eCIRP on the expression of Siglec-G in B-1a cells, in an in vivo experiment we injected mice with rmCIRP intraperitoneally and after 20 h of rmCIRP injection, we assessed the frequency and number of Siglec-G positive B-1a cells in the peritoneal cavity. We found that following intraperitoneal injection of rmCIRP, the frequency and number of Siglec-G positive PerC B-1a cells were significantly decreased by 34.4%, and 62.7%, respectively, as compared to PBS-injected mice (Fig. 4A, B). In an in vitro study, we treated PerC B-1a cells isolated from WT healthy mice with rmCIRP and then assessed the frequency of Siglec-G positive B-1a cells. We found that treatment of murine PerC B-1a cells with rmCIRP significantly decreased the frequency of Siglec-G positive B-1a cells by 20.3% compared to PBS-treated B-1a cells (Fig. 4C). These data suggest that eCIRP directly reduces Siglec-G expression in B-1a cells both in vivo and in vitro conditions.

### Loss of Siglec-G in PerC B-1a cells causes increased expression of IL-6

**Fig. 5:**
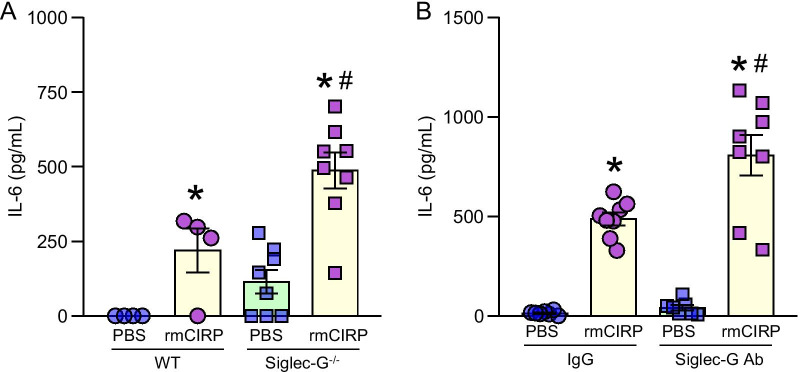
An absence of Siglec-G results in greater production of IL-6 by B-1a cells in response to rmCIRP than WT cells. **A** PerC B-1a cells were collected from WT and Siglec-G^−/−^ mice and stimulated in vitro with rmCIRP or PBS vehicle for 24 h. Supernatant was assessed by ELISA for IL-6 production. Data are expressed as means ± SE (n = 4–8 mice/group) and compared by one-way ANOVA and Student–Newman–Keuls (SNK) method (*p < 0.05 vs. WT PBS treated mice and #p < 0.05 vs. WT rmCIRP treated mice). **B** Blocking Siglec-G by its neutralizing Abs increases IL-6 production following rmCIRP treatment. PerC B-1a cells were collected from WT mice and pretreated with Siglec-G blocking Ab or IgG control. The cells were stimulated in vitro with rmCIRP or PBS vehicle for 24 h. Supernatant was assessed by ELISA for IL-6 production. Data are expressed as means ± SE (n = 8 mice/group) and compared by one-way ANOVA and Student–Newman–Keuls (SNK) method (*p < 0.05 vs. PBS IgG treated mice and ^#^p < 0.05 vs. rmCIRP IgG treated mice)

To determine whether Siglec-G’s deficiency augments inflammatory response in eCIRP-induced PerC B-1a cells, murine B-1a cells isolated from WT and Siglec-G^−/−^ mice were treated with rmCIRP and the supernatant was assessed for the release of IL-6. We found that the treatment of WT mice PerC B-1a cells with rmCIRP significantly increased the release of IL-6 as compared to PBS-treated WT B-1a cells. Interestingly, treatment of Siglec-G^−/−^ PerC B-1a cells with rmCIRP produced significantly higher amount of IL-6 by 223% compared to rmCIRP-treated WT PerC B-1a cells (Fig. 5A). We next adopted a pharmacological approach by blocking/targeting Siglec-G in B-1a cells using anti-Siglec-G Ab and isotype IgG as control. We found that pre-treatment of PerC B-1a cells with anti-Siglec-G Ab significantly increased the release of IL-6 compared to the isotype IgG control pre-treated PerC B-1a cells after stimulation of the cells with rmCIRP (Fig. 5B). These data suggest that genetic deletion and pharmacologic neutralization of Siglec-G result in significant increase of the production of IL-6 from PerC B-1a cells stimulated with rmCIRP.

### Co-culture of Siglec-G neutralized B-1a cells with macrophages releases more cytokines

**Fig. 6:**
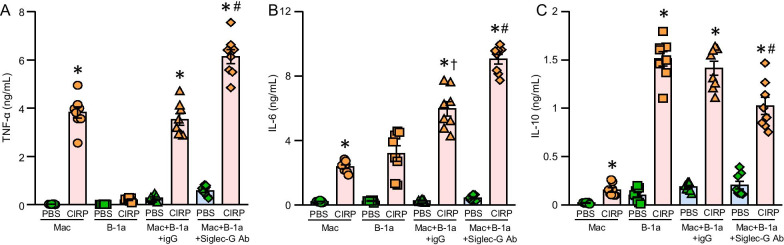
Neutralizing Siglec-G in B-1a cells causes more TNF-α, and IL-6 and less IL-10 production in rmCIRP-treated macrophage co-cultures. PerC macrophages were co-cultured with B-1a cells that had been pretreated with Siglec-G blocking Ab or IgG control and then stimulated with rmCIRP or PBS vehicle for 24 h. Supernatant **A** TNF-α, **B** IL-6, and **C** IL-10 concentration were assessed with ELISA. Data are expressed as means ± SE (n = 8 mice/group) and compared by one-way ANOVA and Student–Newman–Keuls (SNK) method (*p < 0.05 vs. PBS stimulated macrophages, ^#^p < 0.05 vs. macrophages cultured with IgG treated B-1a cells stimulated with rmCIRP, ^†^p < 0.05 vs. untreated B-1a cells stimulated with rmCIRP)

B-1a cells are known to regulate macrophage’s pro-inflammatory function by decreasing the expression of pro-inflammatory cytokines through B-1a cell-released anti-inflammatory cytokine IL-10. To determine the impact of Siglec-G’s deficiency in PerC B-1a cells to alter their immunoregulatory function towards decreasing pro-inflammatory cytokines from macrophages, we first blocked Siglec-G on PerC B-1a cells, washed them to get rid of any unbound Siglec-G neutralizing Abs, and then co-culture them with macrophages in the presence or absence of rmCIRP. We found that blocking Siglec-G in PerC B-1a cells with anti-Siglec-G Ab significantly increased the production of TNF-α and IL-6 in macrophage co-culture system by 173%, and 152%, respectively, compared to rmCIRP-stimulated macrophage co-culture with IgG-treated PerC B-1a cells (Fig. 6A, B). Conversely, we also found that blocking Siglec-G in PerC B-1a cells with anti-Siglec-G Ab significantly decreased the production of IL-10 in the culture supernatant of macrophage co-culture system by 27.5% compared to rmCIRP-stimulated macrophage co-culture with IgG-treated PerC B-1a cells (Fig. 6C). These data suggest that blocking Siglec-G on B-1a cells abrogates their immunoregulatory function towards macrophages by their decreased production of anti-inflammatory cytokine IL-10.

## Discussion

In the current study, we identified that eCIRP levels in the peritoneal cavity were elevated in sepsis, which led us to focus on eCIRP’s role on B-1a cells in sepsis. Treatment of mice with rmCIRP significantly reduced the PerC B-1a cell pool, while in CIRP deficient mice with sepsis there was significantly less reduction of PerC B-1a cell pool as compared to WT mice. These data implicated eCIRP’s role in decreasing PerC B-1a cell pool during sepsis. We also deduced that eCIRP decreased the expression of Siglec-G in B-1a cells. The loss of Siglec-G in eCIRP-treated B-1a cells results in them shifting to a pro-inflammatory phenotype as they produced increased levels of IL-6. We further identified that due to the blockade of Siglec-G on B-1a cells by using Siglec-G neutralizing Abs, their immunoregulatory function on controlling pro-inflammatory cytokine production by the macrophages became attenuated. Collectively, these data suggest that eCIRP not only decreases PerC B-1a cell pool but also hinders B-1a cell’s immunoregulatory functions in sepsis. In our previous study, we showed that the CIRP^−/−^ mice had significantly improved survival rate than the WT sepsis mice (Qiang 2013). In the context of Siglec-G^−/−^ mice study in sepsis, Chen et al.demonstrated that survival rates of Siglec-G^−/−^ mice were significantly declined compared to WT mice (Chen 2011). Thus, targeting eCIRP and restoring Siglec-G expression provide protective outcomes in sepsis.

Given the critical impact of DAMPs in sepsis (Denning et al. 2019), our data of eCIRP-mediated reduction of B-1a cell numbers in the peritoneal cavity directs a new pathophysiological avenue for identifying the possible impacts of other DAMPs like HMGB1, H3, and ATP on B-1a cells in sepsis. Consistent with the finding of eCIRP-induced decrease of PerC B-1a cell pool in sepsis, previous studies have also revealed that intraperitoneal injection of LPS in mice decreases B-1a cell pool in the peritoneal cavity. While intracellular CIRP has been implicated in cell survival and cell proliferation during stress (Liao et al. 2017), extracellular CIRP induces apoptosis in sepsis and ALI (Khan et al. 2017). Extracellular CIRP has been shown to induce endothelial cell pyroptosis (Yang 2016), a process of inflammatory cell death, yet the role of eCIRP in immune-reactive cells’ pyroptosis remains unknown. Although sepsis induces apoptosis, B-1a cells have been shown to be resistant to apoptosis in a radiation-induced injury model. This is attributed to the B-1a cells ability to self-renew via constitutively active STAT3 signal transduction (Otero et al. 2006; Guimarães-Cunha et al. 2016). Therefore, the decrease in PerC B-1a cell pool could be facilitated through other events/factors, rather than apoptosis. We have recently shown that following induction of sepsis, B-1a cells in the peritoneal cavity migrate to the spleen and lymph nodes. A recent study showed that injection of bacteria or LPS into the peritoneal cavity promoted PerC B-1a cells to migrate toward the spleen where they were transformed into plasma cells to produce more immunoglobulin. Migration of PerC B-1 cells has been shown to be associated with the upregulation of CXCR4 as well as an increased migratory response to the chemotactic factor CXCL12 (Moon et al. 2012). Another study revealed that the B-cell-specific loss of CXCR4 decreases B-1a number and IgM production within the bone marrow (BM), a niche of natural IgM, resulting in decreased plasma IgM (Upadhye 2019). As such, it is possible that depending on the expression of CXCR4 on B-1a cells, they may migrate to the BM. Future studies of the expression of CXCR4 on B-1a cells following eCIRP stimulation may provide deep insight into the mechanism of B-1a cell depletion from the peritoneal cavity during sepsis.

Siglec-G is expressed in B-1a cells, as well as in myeloid cells (Royster et al. 2021). NF-κB is constitutively activated in Siglec-G^−/−^ B-1a cells (Ding 2007). Since NF-κB governs the expression of pro-inflammatory cytokines, studies have demonstrated that NF-κB inhibitors protect animals from sepsis (Liu and Malik 2006). In DCs, Siglec-G hinders DAMP mediated effects on NF-κB activation (Chen et al. 2009). In myeloid cells, Siglec-G causes SHP2 and Cbl-dependent ubiquitylation and proteasomal degradation of RIG-I, resulting in a dampening of the type I IFN response (Chen 2013). Given the decreased activation of NF-κB and type-I IFN by Siglec-G, sepsis-induced hyper inflammation can be controlled. Relevant to these above findings, our results demonstrating the increased release of IL-6 by the rmCIRP stimulated Siglec-G^−/−^ B-1a cells or anti-Siglec-G Ab-treated B-1a cells demonstrate the important immunoregulatory role of Siglec-G in B-1a cells. Chen et al*.* showed that Siglec-G-mediated inhibition of HMGB1-induced TLR4 signaling to promote the expression of pro-inflammatory cytokines in macrophages was not dependent on the direct binding of HMGB1 to Siglec-G (Chen 2011). HMGB1 binds to CD24, and this di-molecular complex further binds to Siglec-G through CD24’s sialic acid moiety to form a tri-molecular complex. Siglec-G leads to SHP1 activation, which downregulates NF-κB activity and pro-inflammatory cytokine production (Chen 2011). Our study could not detect the interaction between eCIRP and Siglec-G (data not shown), indicating that Siglec-G-mediated inhibition of eCIRP-induced inflammation is independent of its binding Siglec-G, suggesting the involvement of other factors which may need to be focused on in future studies. The role of Siglec-G in polymicrobial sepsis was identified by using Siglec-G^−/−^ mice, which showed increased susceptibility to sepsis-induced death (Chen et al. 2009). Bacterial sialidases lead to the removal of sialic acids from various ligands of Siglec-G, augmenting inflammation in sepsis (Chen 2011). Conversely, the use of sialic acid coated nanoparticles has been shown to provide beneficial outcomes in septic mice, further lending credence to Siglec-G as a clinically important immunoregulatory molecule (Royster et al. 2021; Spence et al. 2015). In myeloid cells, Siglec-G acts as a negative regulator of DAMPs. CD24 is capable of binding to multiple different DAMPs, which then, in turn, binds to Siglec-G forming a trimeric complex. The DAMP-CD24-Siglec-G trimer then acts to repress DAMP mediated inflammatory responses by activating Siglec-G (Chen et al. 2009). Irradiation causes reduced expression of Siglec-G at its protein level in immune cells, which leads to DAMP-mediated inflammatory responses (Toubai 2014). Irradiation is known to disrupt the intestinal barrier, this results in the bacterial transmigration of intestinal flora into the circulation, resulting in sepsis. The findings of decreased expression of Siglec-G in immune cells in the irradiation model could be mimicked with a polymicrobial sepsis model.

Under sham condition, both WT and CIRP^−/−^ mice had similar contents of Siglec-G^+^ B-1a cells in the peritoneal cavity, but following induction of sepsis by CLP operation, in WT mice, the decrease in Siglec-G^+^ B-1a cells in PerC were significantly higher than the CIRP^−/−^ mice PerC B-1a cells. CIRP is an intracellular RNA chaperon that resides inside the cells under sham condition, but CIRP is released extracellularly to induce inflammation during sepsis. Here, we showed that eCIRP released during sepsis directly reduces Siglec-G expression on B-1a cells. Since septic WT mice release eCIRP, but not septic CIRP^−/−^ mice, we think the significant difference in Siglec-G^+^ B-1a cells in the PerC could be attributed to the effect of eCIRP. To reveal how eCIRP decreases Siglec-G expression in B-1a cells, we found no interaction between eCIRP and Siglec-G (data not shown), indicating that eCIRP-mediated inhibition of Siglec-G expression is independent of its binding to Siglec-G and autoregulation of Siglec-G expression. We previously identified TLR4 as the receptor of eCIRP (Qiang 2013), and the previous study showed that B-1a cells express TLR4 (Rauch 2012). Based on these findings, to determine the mechanism of eCIRP’s direct effect on inhibition of Siglec-G expression in B-1a cells, we treated B-1a cells isolated from WT and TLR4^−/−^ mice with rmCIRP and found that the inhibition of Siglec-G expression in B-1a cells of TLR4^−/−^ mice was less as compared to WT mice B-1a cells, indicating that eCIRP-mediated inhibition of Siglec-G expression occurs through the TLR4 pathway (Additional file 1: Fig. S1).

Human B cell populations change quantitatively and qualitatively in the elderly. A recent work showed the percentage of B-1a cells and their ability to spontaneously secrete IgM decreased with age, which might impact the quality of life and onset of disease within the elderly population (Rodriguez-Zhurbenko et al. 2019). The human findings of age-related decline in natural IgM function has also been proved in elderly mice with pneumococcal infection (Holodick et al. 2016). Natural IgM secreted from B-1a cells plays a wide range of physiological functions (Grönwall et al. 2012). Murine B-1a cell natural IgM is characteristically repertoire skewed, low affinity, and polyreactive (Aziz et al. 2015). B-1a cell-derived natural IgM are able to recognize phosphorylcholine (PC), a prominent component of the cell wall of Gram positive bacteria. Natural IgM can also bind to the membranes of other bacterial pathogens, oxidized low-density lipoprotein (OxLDL), and apoptotic cells (Aziz et al. 2015; Royster et al. 2021). Interaction of natural IgM with an infectious agent can act either by direct neutralization, complement activation, or opsonization leading to phagocytosis and/or Ab-dependent cell-mediated cytotoxicity. In B-1a cells, Siglec-G plays a critical role in BCR-mediated B cell activation and development (Poe and Tedder 2012). Siglec-G-deficient mice have been found to have greatly expanded B-1a cell pools as well as higher titers of natural IgM (Kyaw et al. 2012). However, it is currently unknown if the IgM released from Siglec-G knockout B-1a cells will have the same repertoire skew, affinity, and or polyreactivity as natural IgM from wild-type B-1a cells. Conversely, in the murine sepsis model, Siglec-G knockout mice have been shown to become susceptible to inflammation and mortality (Chen et al. 2009).

## Conclusions

Our study demonstrated novel pathophysiology of eCIRP as mediated by reducing the contents of a regulatory subset of B cells in the peritoneal cavity and converting their immunoregulatory role towards pro-inflammatory in sepsis. We have demonstrated that preservation of Siglec-G on the B-1a cells maintains the cells in an immunoregulatory state, rather than shifting them toward a proinflammatory state. Thus, by targeting eCIRP, the homing, phenotype, and function of B-1a cells can be preserved, ultimately giving rise to beneficial outcomes in sepsis. The phenotype of human B-1a cells has been known (Griffin et al. 2011). In human sepsis patients, increased eCIRP levels are associated with poor outcomes of the disease (Zhou 2015). Future research on determining eCIRP’s role in human B-1a cells in sepsis is warranted.

## Supplementary Information

**Additional file 1: Figure S1.** Treatment with rmCIRP downregulates Siglec-G expression by WT B-1a cells, but not TLR4^−/−^ B-1a cells in vitro. PerC B-1a cells isolated from WT or TLR4^−/−^ mice (Kind gift of Dr. Kavin Tracey of the Feinstein Institutes) were stimulated with rmCIRP in vitro for 24 h. Cells were stained with Siglec-G Ab and subjected to flow cytometric analysis. Frequency of Siglec-G expression on B-1a cells after stimulation with rmCIRP are shown. Data are expressed as means ± SE (n = 3–4 mice/group). The groups were compared by one-way ANOVA and Student–Newman–Keuls (SNK) method (*p < 0.05 vs. PBS-treated WT B-1a cells; #p < 0.05 vs 5 µg/mL rmCIRP-treated WT B-1a cells).

## Data Availability

All data were presented in the manuscript and these are readily available to the readers. This manuscript does not contain additional data.

## Abbreviations

eCIRP: Extracellular cold-inducible RNA-binding protein
PAMPs: Pathogen-associated molecular patterns
DAMPs: Damage-associated molecular patterns
Siglec-G: Sialic acid-binding immunoglobulin-type lectin-G
GM-CSF: Granulocyte–macrophage colony stimulating factor
BCR: B cell receptor
ALI: Acute lung injury
NFATc1: Nuclear factor of activated T-cells, cytoplasmic 1
NF-κB: Nuclear factor-κB
TLR4: Toll-like receptor 4
TREM-1: Triggering receptor expressed on myeloid cells-1
PerC: Peritoneal cavity
HMGB1: High-mobility group box 1
ITIM: Immunoreceptor tyrosine-based inhibition motif
SHP1: Src homology region 2 domain-containing phosphatase 1
RIG-I: Retinoic acid-inducible gene-I
OxLDL: Oxidized low-density lipoprotein
