# Supplementary figures and images for: Extracellular CIRP decreases Siglec-G expression on B-1a cells skewing them towards a pro-inflammatory phenotype in sepsis
Source: Mol Med. 2021 May 31;27:55. doi: 10.1186/s10020-021-00318-y (PMC8165807; doi:10.1186/s10020-021-00318-y)

Supplemental Figure 1

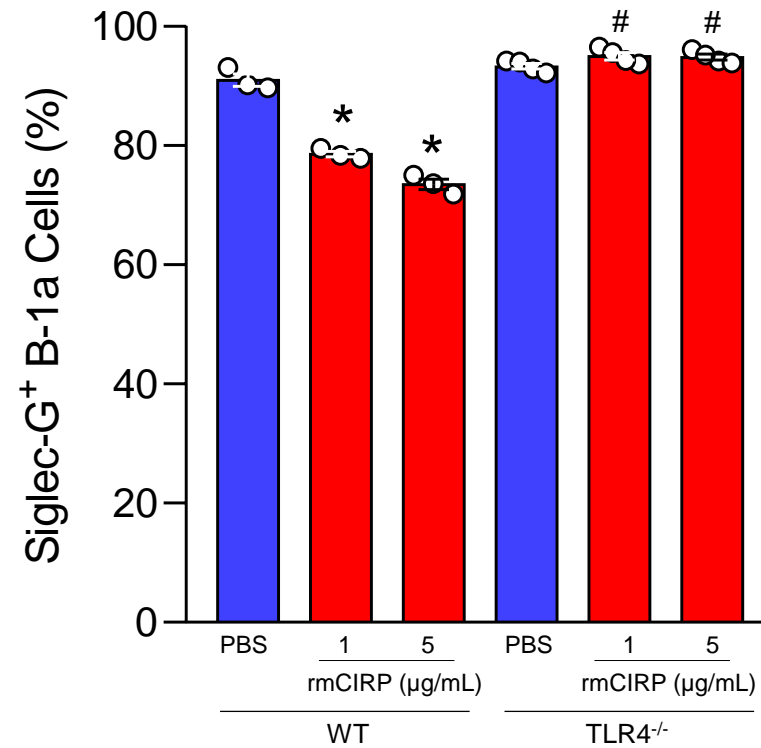

Supplement: Supplementary file 1 — Additional file 1: Figure S1. Treatment with rmCIRP downregulates Siglec-G expression by WT B-1a cells, but not TLR4−/− B-1a cells in vitro. PerC B-1a cells isolated from WT or TLR4−/− mice (Kind gift of Dr. Kavin Tracey of the Feinstein Institutes) were stimulated with rmCIRP in vitro for 24 h. Cells were stained with Siglec-G Ab and subjected to flow cytometric analysis. Frequency of Siglec-G expression on B-1a cells after stimulation with rmCIRP are shown. Data are expressed as means ± SE (n = 3–4 mice/group). The groups were compared by one-way ANOVA and Student–Newman–Keuls (SNK) method (*p < 0.05 vs. PBS-treated WT B-1a cells; #p < 0.05 vs 5 µg/mL rmCIRP-treated WT B-1a cells). [file 10020_2021_318_MOESM1_ESM.pdf]
